# Prognostic nomogram based on the gamma-glutamyl transpeptidase-to-platelet ratio for patients with compensated cirrhotic hepatocellular carcinoma after local ablation

**DOI:** 10.3389/fonc.2024.1406764

**Published:** 2024-07-11

**Authors:** Wenying Qiao, Jiashuo Li, Peiyi Wang, Yuanyuan Zhang, Ronghua Jin, Jianjun Li

**Affiliations:** ^1^ Hepatic Disease and Oncology Minimally Invasive Interventional Center, Beijing You’an Hospital, Capital Medical University, Beijing, China; ^2^ Beijing Ditan Hospital, Capital Medical University, Beijing, China; ^3^ Changping Laboratory, Beijing, China; ^4^ Qingdao Agricultural University, Qingdao, China; ^5^ Beijing Key Laboratory of Emerging Infectious Diseases, Institute of Infectious Diseases, Beijing Ditan Hospital, Capital Medical University, Beijing, China; ^6^ Beijing Institute of Infectious Diseases, Beijing, China; ^7^ National Key Laboratory of Intelligent Tracking and Forecasting for Infectious Diseases, Beijing, China

**Keywords:** hepatocellular carcinoma (HCC), compensated cirrhosis, overall survival (OS), nomogram, gamma-glutamyl transpeptidase-to-platelet ratio (GPR)

## Abstract

**Background:**

Hepatocellular carcinoma (HCC) patients with compensated cirrhosis typically face a high prevalence and unfavorable prognosis. However, there is currently a deficiency in prediction models to anticipate the prognosis of these patients. Therefore, our study included the Gamma-glutamyl transpeptidase-to-platelet ratio (GPR) in analysis and aimed to develop a nomogram for HCC patients with compensated cirrhosis after local ablation.

**Methods:**

Enrolling 669 patients who underwent local ablation at Beijing You’an Hospital during the period from January 1, 2014, to December 31, 2022, this study focused on individuals with compensated cirrhotic HCC. In a ratio of 7:3, patients were allocated to the training cohort (n=468) and the validation cohort (n=201). Lasso-Cox regression was employed to identify independent prognostic factors for overall survival (OS). Subsequently, a nomogram was constructed using these factors and was validated through receiver operating characteristic (ROC) curves, calibration curves, and decision curve analysis (DCA).

**Results:**

GPR, age, and hemoglobin were identified by Lasso-Cox regression as independent prognostic factors of the nomogram. The area under the ROC curves (AUCs) for 3-, 5-, and 8-year OS (0.701, 0.755, and 0.768 for the training cohort; 0.684, 0.707, and 0.778 for the validation cohort), and C-indices (0.695 for training cohort; 0.679 for validation cohort) exhibited the excellent predictive ability of the nomogram. Calibration curves and DCA curves indicated favorable calibration performance and clinical utility. Patients were further stratified into two risk groups according to the median nomogram score. There existed an obvious distinction between the two groups both in the training cohort and validation cohort.

**Conclusion:**

In summary, this research established and validated a novel nomogram to predict OS, which had good predictive power for HCC patients with compensated cirrhosis after local ablation.

## Introduction

Hepatocellular carcinoma (HCC), accounting for more than 90% of primary liver cancer cases, poses a significant threat to human health (1, 2). Based on statistical data, HCC ranks as the sixth most common cancer and the third leading cause of cancer-related death globally (3). Liver cirrhosis stands as a major risk factor for the development of HCC, with 80-90% of detected HCC cases occurring in cirrhotic patients (4, 5). There are approximately 112 million cases of compensated cirrhosis worldwide based on the latest Global Burden of Disease report in 2017, with an annual HCC incidence of 2.2-5% (6, 7). Considering the high prevalence and poor prognosis of compensated cirrhosis in HCC patients, prognostic models to predict overall survival are crucial for these individuals.

Local ablation is considered as a first-line treatment for patients with early-stage HCC, and patients’ survival has improved due to the advances of this therapeutic modality these years (8). As a minimally invasive approach, it has advantages such as small incisions, and less trauma, bleeding, and complications (9). However, the prognosis of HCC is still unsatisfactory after ablation, with a 5-year overall survival of 30-40% (10, 11). Therefore, our study focused on the patients who received local ablation.

It has been widely acknowledged that the inflammatory response, which reflects cancer status and affects the progression of tumors, is apparently interconnected with the tumor microenvironment (12). Gamma-glutamyl transpeptidase (GGT)-to-platelet (PLT) ratio (GPR), an inflammatory marker, was proposed in the past few years as an innovative predictor for liver disease (13). Numerous studies have substantiated the predictive capability of GPR in predicting cirrhosis in patients with hepatitis B virus (HBV) infection and recognized it as an independent prognostic factor for HCC (14, 15). A study by Yang et al. observed that the specific value of GPR was selected as 0.30, which had favorable efficiency for distinguishing high-risk patients (16). Nevertheless, as for HCC patients with compensated cirrhosis, the predictive capability of GPR for prognosis remains uncertain. Additionally, there is also lacking effective nomograms to predict patients’ mortality rates.

Therefore, this study included GPR in the analysis and investigated its association with overall survival. Subsequently, a nomogram for HCC patients with compensated cirrhosis after local ablation was built based on demographic and clinical variables selected by Lasso-Cox regression. Moreover, the comparison of survival times among different risk groups derived from the established nomogram was done, aiming to facilitate the identification of high-risk populations and offer more precise clinical guidance.

## Methods

### Patients

We retrospectively analyzed the clinical data of HCC patients with compensated cirrhosis who received ablation therapy at Beijing You’an Hospital affiliated with Capital Medical University between January 1, 2014, and December 31, 2022. A total of 669 patients were included in the final analysis and these patients were randomly assigned to two groups in a ratio of 7:3, with 468 patients in the training set and 201 in the validation set (**Supplementary Figure S1**). This study obtained approval from the Ethics Committee of Beijing You’an Hospital affiliated with Capital Medical University, and informed consent was waived due to the retrospective nature of the study.

Patients were diagnosed with HCC and cirrhosis according to the American Association for the Study of Liver Disease (AASLD) and the European Association for the Study of the Liver (EASL) (17, 18). The early-stage of HCC was defined according to Barcelona Clinic Liver Cancer (BCLC) stage and Child-Pugh stage (19, 20). Compensated cirrhosis was defined as cirrhosis in the absence of clinical complications, including ascites, variceal bleeding, and hepatic encephalopathy.

The inclusion criteria were as follows: (1) age from 18 to 75 years; (2) BCLC stage 0 or A; (3) Child-Pugh stage A; (4) HCC patients with compensated cirrhosis. The exclusion criteria were as follows: (1) incomplete clinical or follow-up data; (2) with distant metastases or other malignancies; (3) insufficiency of vital organs; (4) bacterial or viral infections; (5) rheumatism or blood disorders that can cause platelet changes.

### Data collection and follow-up

Demographics and laboratory data were collected from the electronic patient records, including age, gender, medical history (smoking, drinking, antiviral treatment, hypertension, diabetes mellitus), BCLC stage, tumor number, tumor size, red blood cell (RBC), hemoglobin (Hb), white blood cell (WBC), alanine aminotransferase (ALT), aspartate transaminase (AST), alkaline phosphatase (ALP), total bilirubin (TBIL), direct bilirubin (DBIL), neutrophils (Neu), lymphocytes (Lym), monocytes (Mon), albumin (Alb), globulin (Glob), prealbumin (Palb), prothrombin time (PT), thrombin time (TT), activated partial thromboplastin time (APTT), and international normalized ratio (INR). Meanwhile, we included the gamma-glutamyl transpeptidase to platelet ratio (GPR) in our analysis to increase the prognostic value of the proposed nomogram.

A close follow-up of all patients after local ablation was conducted, and it was completed via outpatient consultation or telephone calls. The typical follow-up was performed every 3 months in the first year and then every 6 months thereafter, which comprised physical examination, laboratory tests, and imaging examination. The last day of follow-up was July 1, 2023. Overall survival (OS), as the primary endpoint of this study, was defined as the interval from local ablation to either the occurrence of death or the last follow-up.

### Ablation procedure

All enrolled patients were treated with local ablation, which was performed by qualified hepatologists and interventional radiologists. The specific process includes 5 items: (1) Appropriate position for ablation was determined by computed tomography (CT) or magnetic resonance imaging (MRI). (2) The ablation needle was inserted in the marked skin, and followed by image scanning to track the ablation process. (3) For the purpose of attaining complete ablation, operators should expand the ablative range and contemplate multiple sites, overlapping, or repeated ablation. (4) In order to prevent tumor implantation and postoperative bleeding, the needle track required to be heated in the final stage. (5) Following the ablation, all patients underwent imaging examinations to assess treatment efficacy and possible complications.

The methods of local ablation include radiofrequency ablation (RFA), microwave ablation (MWA), and cryoablation, among others. By introducing radiofrequency electrodes directly into the tumor tissue, RFA utilizes high-frequency electrical currents to generate heat, resulting in necrosis. While MWA uses microwave energy to generate heat and kill tumor cells. The characteristic of microwave energy is their ability to rapidly and evenly heat tissues. As for cryoablation, it utilizes extremely low temperature of medium (such as liquid nitrogen or argon gas) to freeze and destroy tumor cells. These ablation therapeutic modalities have specific advantages in the treatment of cancer, and the specific method was selected by clinical physicians in our study.

### Statistical analysis

Continuous variables were presented as mean and standard deviation (mean ± SD) and analyzed by Student’s t-test or non-parametric test. Categorical variables were expressed as frequency (percentage) and contrasted using the Chi-square test.

In this research, patients were randomly split into the training set (n=468) and validation set (n=201) in a ratio of 7:3. The training cohort was utilized for building the nomogram, while the validation cohort was specifically employed to verify its performance. Concurrently, Lasso regression and multivariate Cox regression analysis were used to determine the independent risk factors associated with OS. Significant factors with p-values below the threshold of 0.05 were included in the nomogram. Subsequently, patients were categorized into low-risk group and high-risk group according to the scores calculated from the nomogram. Kaplan-Meier curves and log-rank tests were then employed to compare OS between the two groups. Additionally, the receiver operating characteristic (ROC) curves were plotted and the area under the ROC curves (AUCs) was calculated to evaluate the discriminative ability. Model calibration and clinical utility were assessed by calibration curves and decision curve analysis (DCA), respectively. At last, we used the ROC curve to observe the predictive ability of GPR and other risk factors.

All analyses were conducted using R statistical software (version 4.1.2) in this study. Statistical significance was set at p<0.05 (two-tailed).

## Results

### Baseline characteristics

During the period from January 1, 2014, to December 31, 2022, a total of 669 HCC patients with compensated cirrhosis after local ablation were recruited in this study and randomized into two groups with a 7:3 ratio. There were 468 patients constituted the training set, while 201 formed the validation set. All patients received ablation therapy without any combined treatments, and there were 150 (22.4%) patients died during the follow-up period. Our study concluded patients’ last follow-up on July 1, 2023, with a median follow-up duration of 52.4 months.

The baseline clinical characteristics of all patients were presented in **Table 1**, which revealed no statistical differences between the two groups (p>0.05). Among these patients, the average age was 56.28 years, with 544 (81.3%) males and 125 (18.7%) females. Notably, 285 (42.6%) patients had a history of smoking and 215 (32.1%) had a history of drinking. Furthermore, 185 (27.7%) individuals were diagnosed with hypertension and 133 (19.9%) with diabetes mellitus. The training and validation cohorts both had a majority of patients in BCLC stage A (71.2% vs. 65.2%), with most solitary tumors (68.4% vs. 71.1%) and tumor size less than 3cm (65.6% vs. 60.1%).

**Table 1:** Clinical characteristics for training and validation cohorts.

Characteristic	Training cohort (N=468)	Validation cohort (N=201)	P value
Age	56.25 ± 9.32	56.35 ± 9.37	0.896
Gender (%)			0.904
Male	380 (81.2)	164 (81.6)	
Female	88 (18.8)	37 (18.4)	
Hypertension (%)			0.649
Yes	127 (27.1)	58 (28.9)	
No	341 (72.9)	143 (71.1)	
Diabetes (%)			0.403
Yes	97 (20.7)	36 (17.9)	
No	371 (79.3)	165 (82.1)	
Antiviral (%)			0.159
Yes	288 (61.5)	112 (55.7)	
No	180 (38.5)	89 (44.3)	
Smoking (%)			0.456
Yes	195 (41.7)	90 (44.8)	
No	273 (58.3)	111 (55.2)	
Drinking (%)			0.060
Yes	140 (29.9)	75 (37.3)	
No	328 (70.1)	126 (62.7)	
BCLC (%)			0.124
0	135 (28.8)	70 (34.8)	
A	333 (71.2)	131 (65.2)	
T.N. (%)			0.477
Single	320 (68.4)	143 (71.1)	
Multiple	148 (31.6)	58 (28.9)	
T.S. (%)			0.251
<3cm	307 (65.6)	141 (70.1)	
≥3cm	161 (34.4)	60 (29.9)	
GPR	0.67 ± 0.72	0.61 ± 0.56	0.284
RBC (10^12/L)	4.31 ± 0.58	4.28 ± 0.47	0.443
Hb (g/L)	134.63 ± 18.95	133.79 ± 16.42	0.584
WBC (10^9/L)	5.29 ± 2.12	5.16 ± 1.90	0.477
Neu (10^9/L)	3.40 ± 1.84	3.23 ± 1.60	0.230
Lym (10^9/L)	1.33 ± 0.64	1.38 ± 0.67	0.354
Mon (10^9/L)	0.42 ± 0.24	0.42 ± 0.22	0.948
ALT (U/L)	31.34 ± 18.33	32.44 ± 18.77	0.478
AST (U/L)	30.48 ± 12.69	31.91 ± 16.27	0.222
ALP (U/L)	84.92 ± 32.39	84.36 ± 29.51	0.834
TBIL (umol/L)	17.16 ± 7.73	16.84 ± 7.39	0.626
DBIL (umol/L)	5.58 ± 3.19	5.44 ± 3.48	0.611
Alb (g/L)	38.20 ± 3.98	38.35 ± 3.80	0.643
Palb (U/L)	150.36 ± 55.56	145.36 ± 52.95	0.280
Glob (g/L)	28.11 ± 4.71	28.09 ± 5.44	0.962
APTT (s)	33.09 ± 4.03	33.09 ± 3.87	0.995
PT (s)	12.35 ± 1.19	12.29 ± 1.28	0.587
TT (s)	15.56 ± 2.10	15.70 ± 2.17	0.465
INR	1.10 ± 0.10	1.09 ± 0.11	0.390

Continuous variables were presented as mean and standard deviation (mean ± SD). Categorical variables were expressed as frequency (percentage).

BCLC, Barcelona Clinic Liver Cancer; T.N., tumor number; T.S., tumor size; GPR, gamma-glutamyl transpeptidase-to-platelet ratio; RBC, red blood cell; Hb, hemoglobin; WBC, white blood cell; Neu, neutrophil; Lym, lymphocyte; Mon, monocyte; ALT, alanine aminotransferase; AST, aspartate aminotransferase; ALP, alkaline phosphatase; TBIL, total bilirubin; DBIL, direct bilirubin; Alb, albumin; Palb, prealbumin; Glob, globulin; APTT, activated partial thromboplastin time; PT, prothrombin time; TT, thrombin time; INR, international normalized ratio.

### Independent prognostic factors for OS

**Figure 1:**
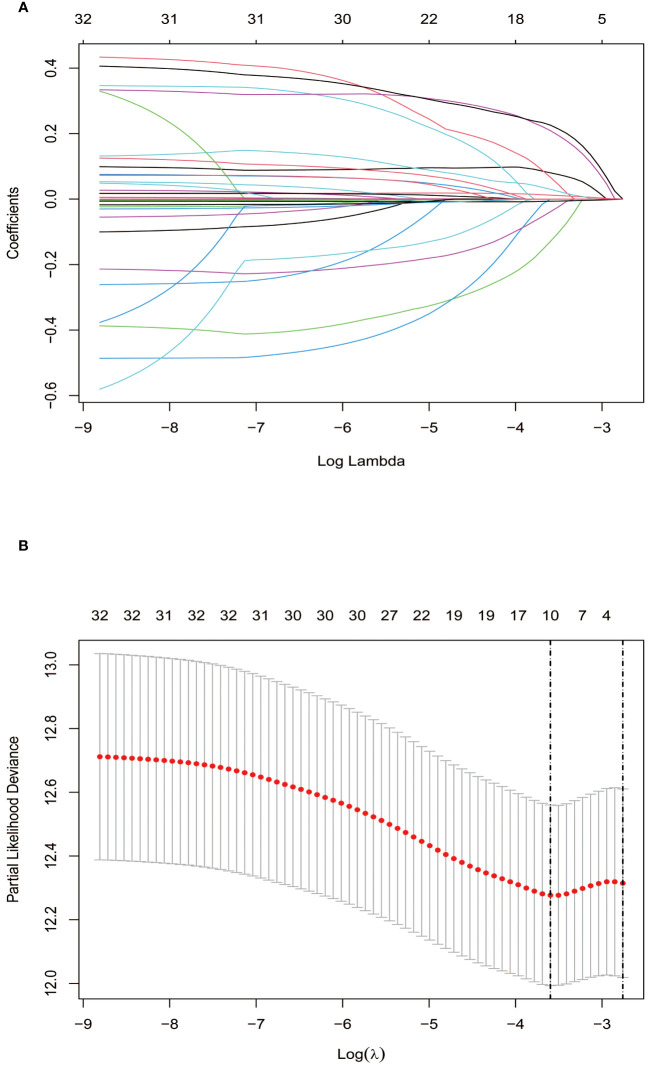
Screening of independent prognostic factors based on Lasso regression. **(A)** The variation characteristics of the coefficient of variables; **(B)** The selection process of the optimum value of the parameter λ by cross-validation method.

Lasso regression, utilizing a loss function with L1 regularization to penalize model coefficients while minimizing the objective function, was employed to screen risk factors associated with OS (**Figure 1**). The 10-fold cross-validation method was applied to select the optimal λ value, which was determined to be 0.0274 (Log λ = -1.562). Significant risk factors filtered by Lasso regression were GPR, age, gender, history of antiviral therapy, history of drinking, tumor number, tumor size, BCLC stage, Hb, and Palb. These variables were further incorporated into the multivariable Cox regression analysis, revealing that GPR (HR:1.257, 95% CI: 1.037-1.527), age (HR:1.025, 95% CI: 1.003-1.048), and Hb (HR:0.988, 95% CI: 0.978-0.999) as the independent prognostic factors for OS (**Table 2**).

**Table 2:** Multivariate Cox regression analysis based on the results of Lasso regression.

Variables	HR (95%CI)	P value
GPR	1.257 (1.037-1.527)	**0.020**
Age	1.025 (1.003-1.048)	**0.024**
Gender	0.654 (0.361-1.186)	0.162
Antiviral	0.826 (0.558-1.222)	0.338
Drinking	1.340 (0.881-2.037)	0.172
BCLC	1.139 (0.608-2.134)	0.845
T.N.	1.421 (0.912-2.214)	0.121
T.S.	1.398 (0.891-2.193)	0.146
Hb	0.988 (0.978-0.999)	**0.031**
Palb	0.998 (0.994-1.002)	0.356

Bolded values indicate a P-value less than 0.05, which represent statistical significance.

HR, hazard ratio; GPR, gamma-glutamyl transpeptidase-to-platelet ratio; BCLC, Barcelona Clinic Liver Cancer; T.N., tumor number; T.S., tumor size; Hb, hemoglobin; Palb, prealbumin.

### Development of the nomogram

**Figure 2:**
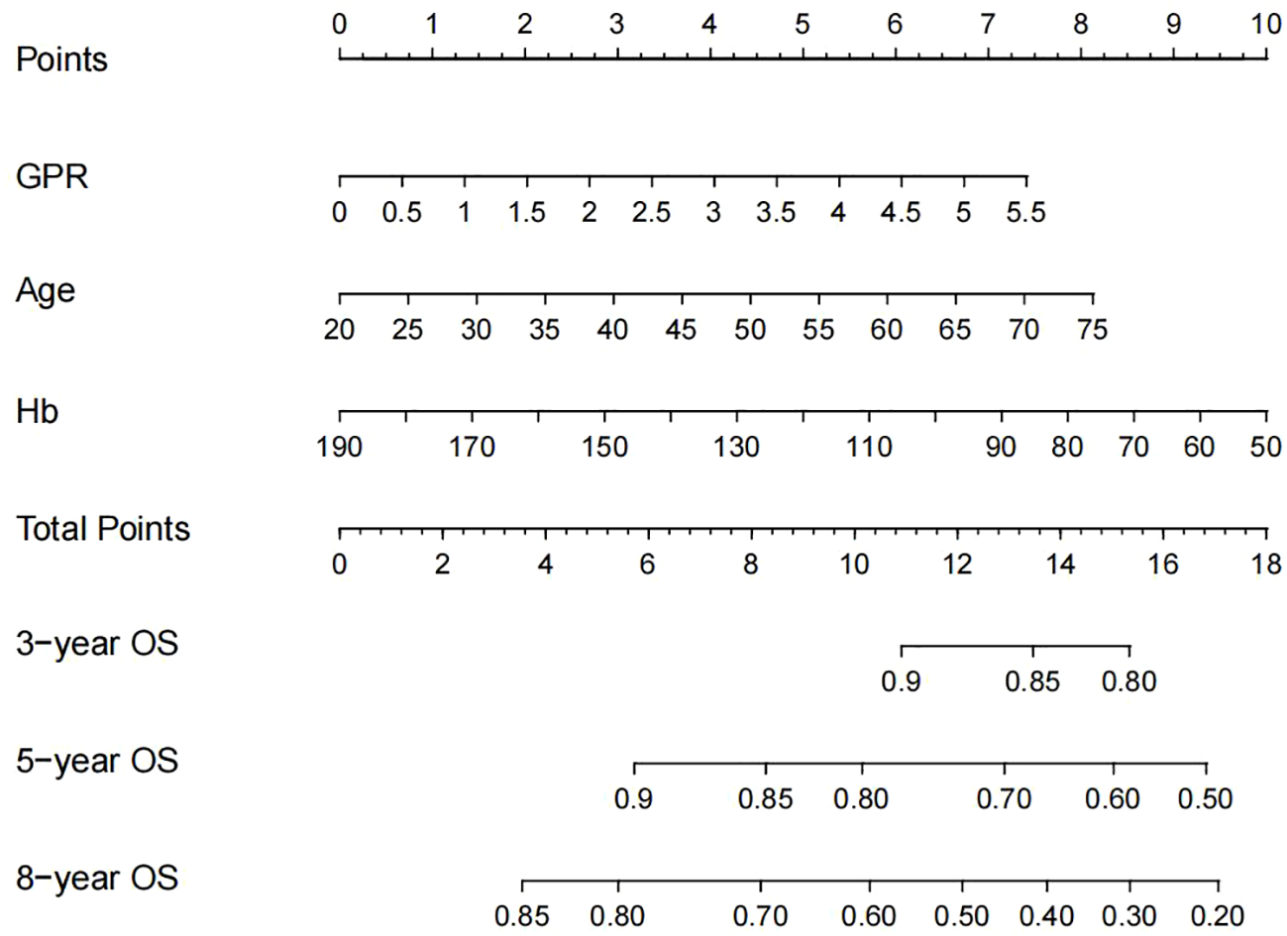
Nomogram, including GPR, age, and Hb for 3-, 5-, and 8-year OS in HCC patients with compensated cirrhosis. GPR, gamma-glutamyl transpeptidase-to-platelet ratio; Hb, hemoglobin; OS, overall survival; HCC, hepatocellular carcinoma.

Based on these independent prognostic factors, we constructed a nomogram for predicting the OS of compensated cirrhotic HCC patients who received local ablation (**Figure 2**). Every risk factor corresponds to a specific score according to its value on the nomogram. It was necessary to sum the scores of factors and draw a vertical line at the corresponding total point. After these steps, the vertical line intersects with three lines representing mortality risk, which forecast the 3-, 5-, and 8-year OS.

**Figure 3:**
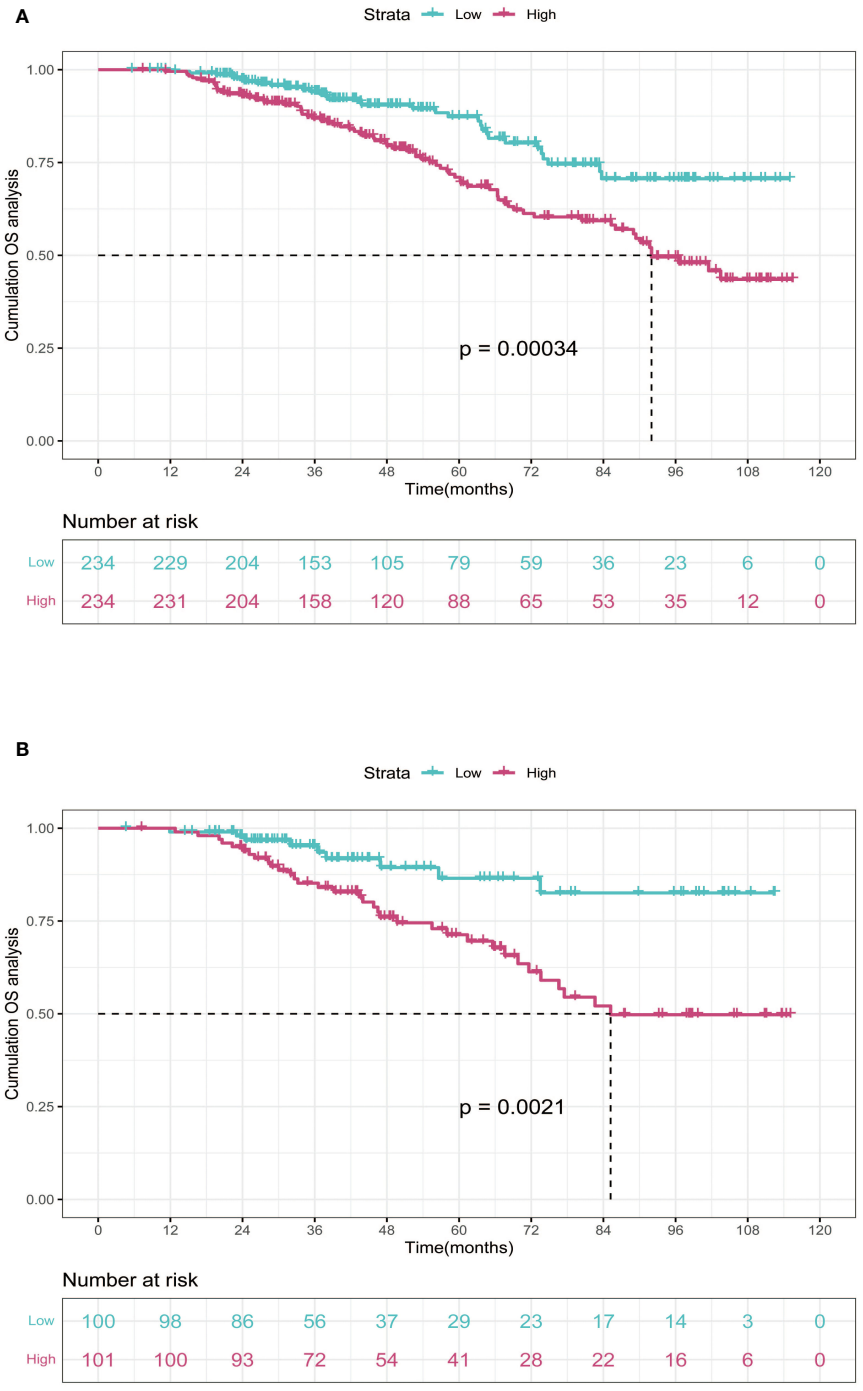
Kaplan-Meier curves of OS for two risk groups classified by the median nomogram score in training and validation cohort. **(A)** training cohort; **(B)** validation cohort. OS, overall survival.

In the training cohort, patients were categorized into low-risk group (n=234) and high-risk group (n=234) in light of the nomogram. The Kaplan-Meier curve was plotted, indicating that the median OS was 92.0 months for the high-risk group, while it was not reached in the low-risk group (**Figure 3**). The cumulative OS rates for 3-, 5-, and 8-year were 86.9%, 71.0%, and 49.5% in the high-risk group, while 94.0%, 87.3%, and 70.6% in the low-risk group. There existed an obvious distinction in OS among the two groups (p=0.00034).

**Figure 4:**
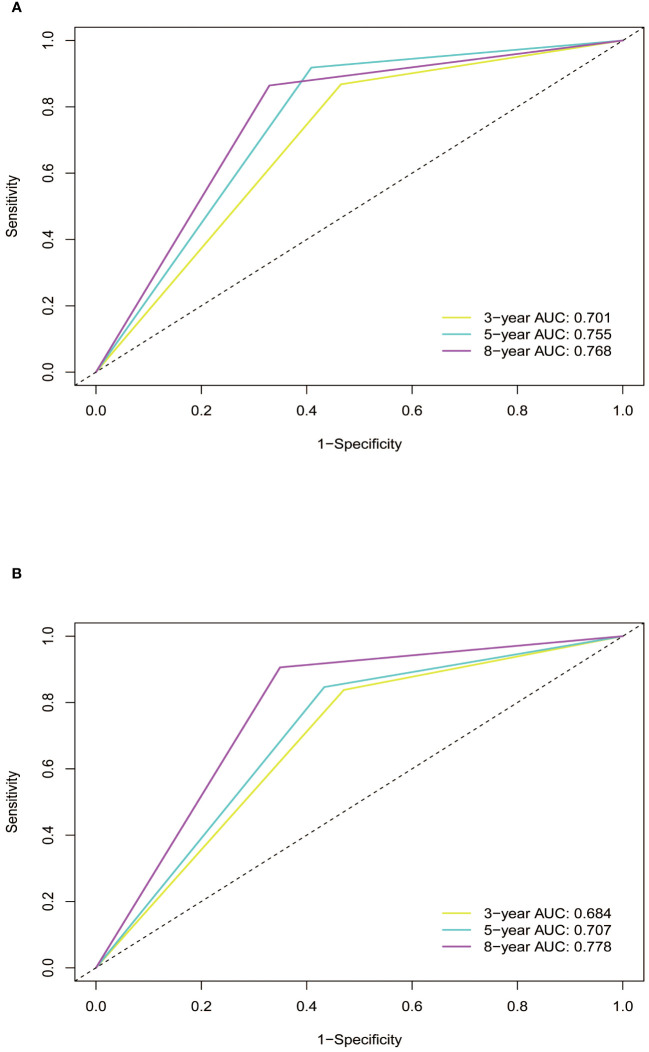
ROC curves of the nomogram in training and validation cohort. **(A)** In the training cohort, the AUCs for 3-, 5- and 8-year OS were 0.701, 0.755, and 0.768, respectively. **(B)** In the validation cohort, the AUCs for 3-, 5- and 8-year OS were 0.684, 0.707, and 0.778, respectively. ROC, receiver operating characteristic curve; AUC, area under the ROC curve; OS, overall survival.

**Figure 5:**
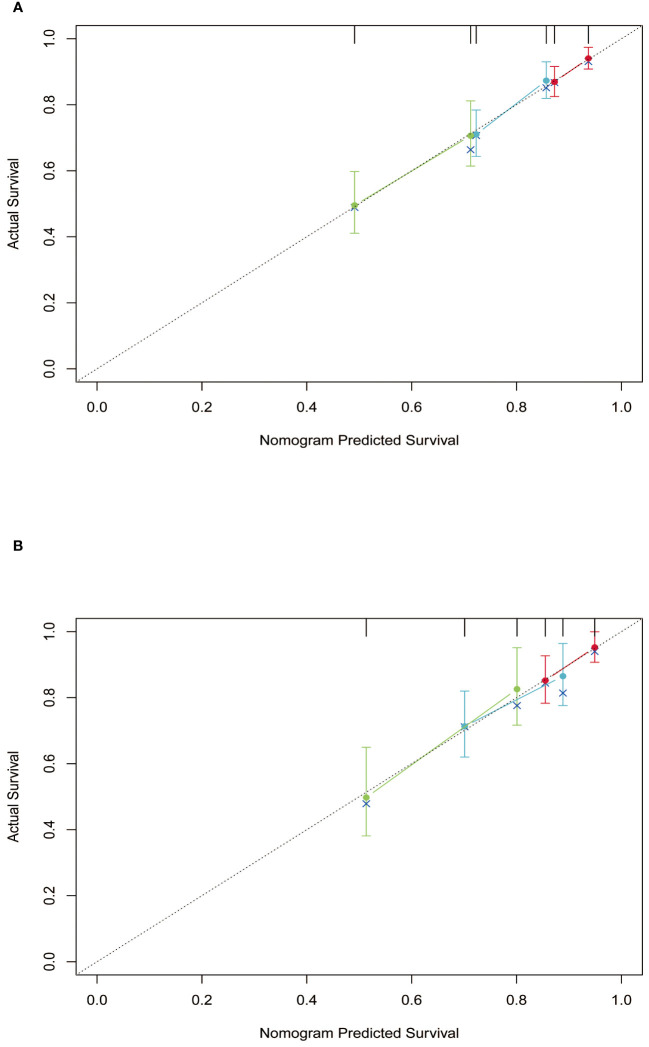
Calibration curves of the nomogram in training and validation cohort. **(A)** training cohort; **(B)** validation cohort.

**Figure 6:**
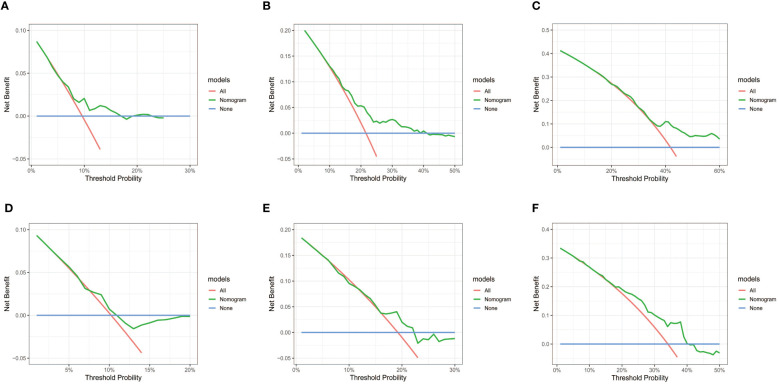
DCA curves of the nomogram in training and validation cohort. **(A-C)** DCA for 3-, 5- and 8-year OS in training cohort. **(D-F)** DCA for 3-, 5- and 8-year OS in validation cohort. DCA, decision curve analysis; OS, overall survival.

Subsequently, the time-dependent ROC curve was drawn and the C-index in the training set was 0.695 (95% CI: 0.656-0.734). It showed that AUCs of 3-, 5-, and 8-year were 0.701, 0.755, and 0.768. The 1-specificity and sensitivity of 3-, 5-, and 8-year were (0.468, 0.862), (0.411, 0.913), and (0.331, 0.861), respectively (**Figure 4**). These outcomes highlighted the advantageous discriminative ability. At last, a calibration curve (**Figure 5**) and DCA curves (**Figure 6**) were created, affirming that the nomogram demonstrated good calibration and clinical utility.

### Validation of the nomogram

In order to further validate the reliability of this nomogram, we performed internal validation in our study. According to the nomogram, patients in the validation cohort were also classified into two groups by the Kaplan-Meier curve: low-risk group (n=100) and high-risk group (n=101) (**Figure 3**). The median OS was 92.0 months for the high-risk group, while it was not reached in the low-risk group. The cumulative OS rates for 3-, 5-, and 8-year were 85.2%, 71.3%, and 49.8% in the high-risk group, while 95.2%, 86.5%, and 82.6% in the low-risk group. In concordance with the training cohort, there was also a statistically significant discrepancy in OS among the two groups (P=0.0021).

The C-index in the validation cohort was 0.679 (95% CI: 0.616-0.742) and the AUCs for 3-, 5-, and 8-year were 0.684, 0.707, and 0.778, which suggested the favorable diagnostic value. The 1-specificity and sensitivity of the ROC curve were (0.472, 0.832), (0.434, 0.844), and (0.351, 0.903) for 3-, 5-, and 8-year (**Figure 4**). The calibration curve exhibited a good match (**Figure 5**), and the DCA curves also had good clinical practicability (**Figure 6**).

### The predictive performance of the GPR

The discriminative ability of the GPR and other risk factors was assessed using ROC curve (**Supplementary Figure S2**). The AUC values were 0.514 for age, 0.504 for hemoglobin, 0.580 for fibrosis 4 score (Fib-4), 0.614 for aspartate transaminase to platelet ratio index (APRI), and 0.652 for GPR, respectively. The outcome indicated that the GPR was observed to have better predictive ability compared with other risk factors. Moreover, we explored the AUC value of the combination of GPR and neutrophil to lymphocyte ratio index (NLR) based score (AUC=0.679), which showed improved discriminability compared to GPR alone. Thus, the predictive ability of GPR combined with NLR need more studies to investigate.

### The predictive performance of the nomogram for DFS

At last, our study used KM curves to analyze the predictive ability of the nomogram for disease-free survival (DFS). It was defined as the time from the date of local ablation to recurrence or last of follow-up. Although it underperformed in the validation cohort, there existed an obvious distinction among training cohort (**Supplementary Figure S3**). In the training cohort, the median DFS was 18.0 months for the high-risk group, and 24.5 months for the low-risk group. The cumulative DFS rates for 1-, 3-, and 5-year were 66.2%, 24.6%, and 16.8% in the high-risk group, while 73.9%, 35.4%, and 26.0% in the low-risk group. These outcomes demonstrated that the nomogram had a certain predictive capability for DFS.

## Discussion

Despite significant progress regarding the treatment of HCC in recent years, the prognosis for this malignancy continues to be unfavorable (21). Liver cirrhosis, especially compensated liver cirrhosis, has a substantial number of patients worldwide (6). It remains a main etiological factor for the development of HCC, which seriously impacts the life quality of patients (22). Thus, our study concentrated on compensated cirrhotic patients with HCC and developed a nomogram for OS to help guide clinical decision-making. The current nomogram exhibited effective predictive performance, as indicated by AUCs, C-indexes, calibration curves, and DCA plots.

The treatment modalities for HCC are diverse and typically based on the patients’ health status and the stage of the tumor. Conventional methods include surgical resection, local ablation, anti-angiogenesis therapy, immunotherapy, and second-line treatments (23). Local ablation, encompassing techniques such as RFA, MWA, and cryoablation, holds a prominent position in the treatment of HCC. The ablation process involves inserting an ablation needle into tumor tissues under the guidance of imaging techniques, and high-frequency radio waves are applied to damage HCC cells (24). Several studies have demonstrated that local ablation provides effective therapy for patients with early-stage HCC during long-term clinical practice experience (25, 26). Nevertheless, the prognosis after ablation remains unfavorable, and deserves further investigation.

In this study, we employed Lasso regression to identify independent prognostic factors and construct a nomogram. This approach regulates the rigor of feature selection by adjusting the regularization parameter λ, facilitating the screening of features, and reducing the dimensionality of the prediction model. It enables more effective exploration of voluminous and complex datasets and somewhat addresses the limitations in overfitting and multicollinearity (27, 28). The nomogram developed by Lasso regression can assist in identifying patients with a high risk of mortality, and effectively improve the prognosis through early intervention. Meanwhile, developing personalized treatment plans based on the different prognoses of HCC patients could efficiently allocate medical resources and achieve precision medicine.

A crucial prognostic factor in the nomogram is GPR, which objectively indicates the combination of coagulation status and liver function. Initially introduced in 2015 by Lemoine et al. for the clinical evaluation of HBV-related hepatic cirrhosis, GPR serves as a more precise routine laboratory marker than the aspartate transaminase-to-platelet ratio index (APRI) and Fib-4 for staging liver fibrosis in patients with chronic HBV infection (13). A retrospective study, involving 182 patients with HBV-associated HCC, investigated the prognostic significance of GPR. The findings revealed that a high level of GPR was associated with unfavorable recurrence-free survival and overall survival (29). Similarly, research by Wang et al. also acknowledged the value of GPR in the prognosis of HCC patients and established a predictive model. As an inflammation-related factor, GPR independently correlated with the survival of HCC patients who underwent hepatectomy (30).

Notably, GGT is a notable enzyme located in cell membranes and actively participates in glutathione metabolism (31). Functioning as a crucial antioxidant, it plays a pivotal role in the protection of liver cells from the detrimental effects of oxidative stress (32). An elevated GGT level not only signals liver damage but also contributes to the development of HCC. Various studies have demonstrated that GGT could induce DNA damage and regulate the cell cycle, highly correlating with tumor progression (33, 34). Platelets are small, nonnuclear fragments of blood cells, which are detached from bone marrow megakaryocytes. When blood vessels are damaged, platelets rapidly gather at the injury site and release platelet-activating factors to promote clot formation (35). It plays an active role in various stages of tumorigenesis, encompassing tumor growth, tumor cell extravasation, and metastasis. Moreover, their secretion of large quantities of microparticles and exosomes helps to effectively coordinate tumor-host crosstalk (36, 37).

Other clinical characteristics in the nomogram include age and hemoglobin. In particular, age is a recognized risk factor for the overall survival. Older patients with HCC generally had unfavorable prognoses due to poor baseline status, high mutation burden, rapid tumor progression, and co-morbidities (38). It is shown in some research that elderly patients exhibit diminished liver weight and portal blood flow rate, leading to weakened liver repairability and a poor prognosis (39). Hemoglobin, a special protein in red blood cells, is a vital carrier responsible for transporting oxygen (40, 41). It is crucial for maintaining normal oxygen supply in the body. The concentration of hemoglobin is the key factor in assessing respiratory health and detecting conditions. Clinical trials have revealed that a majority of cancer patients have low hemoglobin levels as a consequence of the disease. Specifically, reduced hemoglobin has a significant impact on the prognosis through several mechanisms, such as cellular compromise and impaired oxygenation (42).

There are some limitations that should be acknowledged in our study. Firstly, this study was conducted at a singular medical center, and the sample size was insufficient. Moreover, despite internal validation being undertaken, the absence of external validation remains as another notable limitation. Our future studies could benefit from multicenter collaborations to validate the nomogram across diverse patient populations and healthcare settings, enhancing its external validity. And then, the retrospective records of this study, which rely on historical patient information, lead to inevitable bias. This bias occurred because the study depended on existing data, which may exclude undocumented cases that were available for analysis. Finally, the population selected for this study only included HBV-related HCC patients with compensated cirrhosis. Thus, more studies are required to emerge with a solid foundation for the wider application of our nomogram.

## Conclusions

In conclusion, we established and validated a nomogram model incorporating GPR, age, and hemoglobin for predicting the 3-, 5-, and 8-year OS among HCC patients with compensated cirrhosis who underwent local ablation. This nomogram exhibited good predictive capability, which could be instrumental in postoperative surveillance and early intervention.

## Data availability statement

The raw data supporting the conclusions of this article will be made available by the authors, without undue reservation.

## Ethics statement

The studies involving humans were approved by Ethics Committee of Beijing You’an Hospital affiliated with Capital Medical University. The studies were conducted in accordance with the local legislation and institutional requirements. The ethics committee/institutional review board waived the requirement of written informed consent for participation from the participants or the participants’ legal guardians/next of kin because this was a retrospective analysis of existing data.

## Author contributions

WYQ: Data curation, Software, Writing – review & editing. JSL: Data curation, Software, Writing – original draft. PYW: Writing – original draft. YYZ: Conceptualization, Funding acquisition, Methodology, Supervision, Visualization, Writing – review & editing. RHJ: Conceptualization, Funding acquisition, Methodology, Supervision, Visualization, Writing – review & editing. JJL: Conceptualization, Funding acquisition, Methodology, Supervision, Visualization, Writing – review & editing.

## Supplementary material

Supplementary Figure 1 Flow chart of the patients included in the study.

Supplementary Figure 2 The ROC curve of GPR, age, hemoglobin, Fib-4, APRI, and the combination of GPR and NLR. GPR, gamma-glutamyl transpeptidase-to-platelet ratio; Fib-4, fibrosis 4 score; APRI, aspartate transaminase to platelet ratio index; NLR, neutrophil to lymphocyte ratio index.

Supplementary Figure 3 Kaplan-Meier curves of RFS for two risk groups classified by the nomogram in training and validation cohort. **(A)** training cohort; **(B)** validation cohort. RFS, recurrence-free survival.
