# Supplementary material for: Prognostic nomogram based on the gamma-glutamyl transpeptidase-to-platelet ratio for patients with compensated cirrhotic hepatocellular carcinoma after local ablation
Source: Front Oncol. 2024 Jul 11;14:1406764. doi: 10.3389/fonc.2024.1406764 (PMC11269228; doi:10.3389/fonc.2024.1406764)
Supplement: Supplementary Figure 1 — Flow chart of the patients included in the study. [file DataSheet_1.docx]

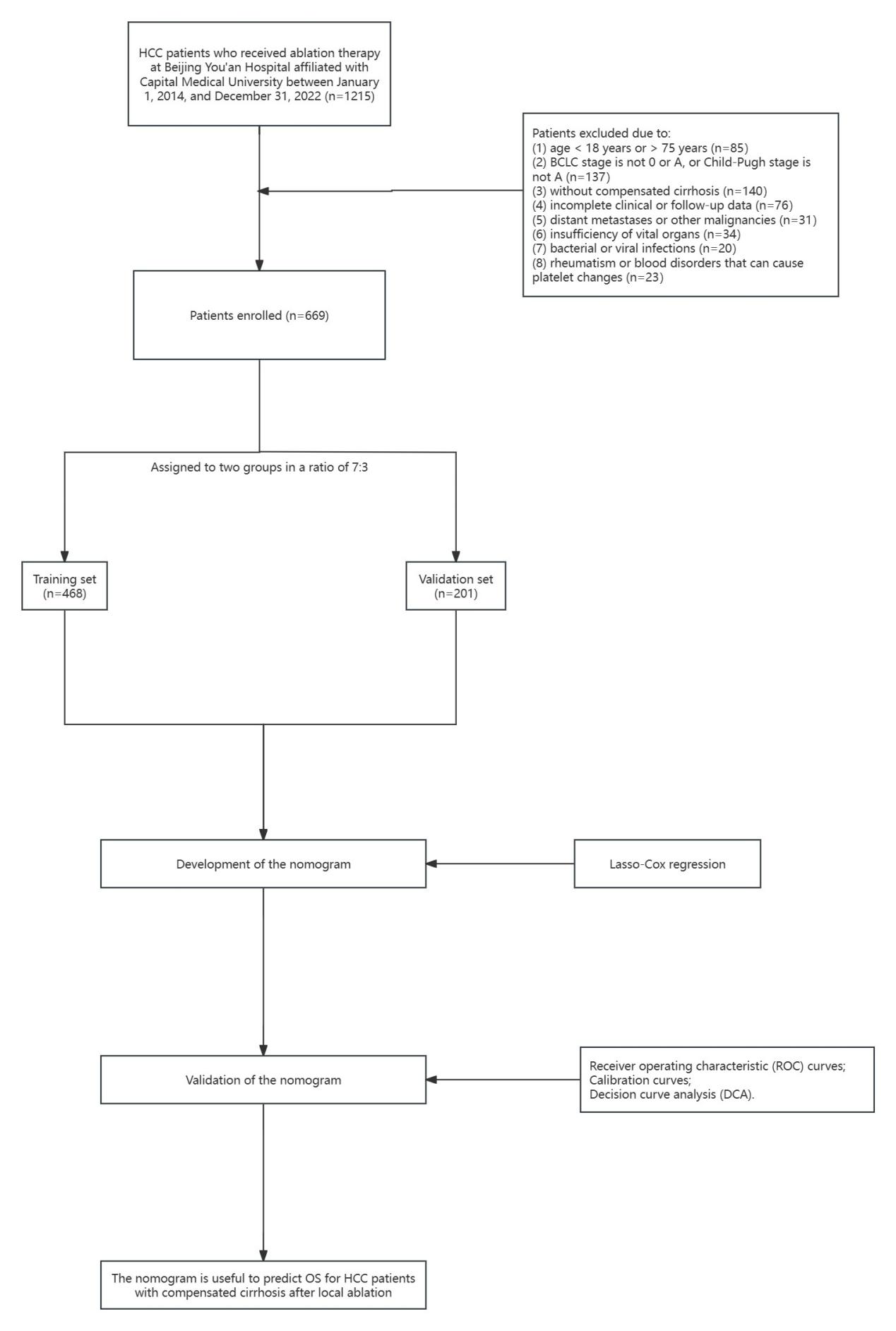


**Figure S1** Flow chart of the patients included in the study.


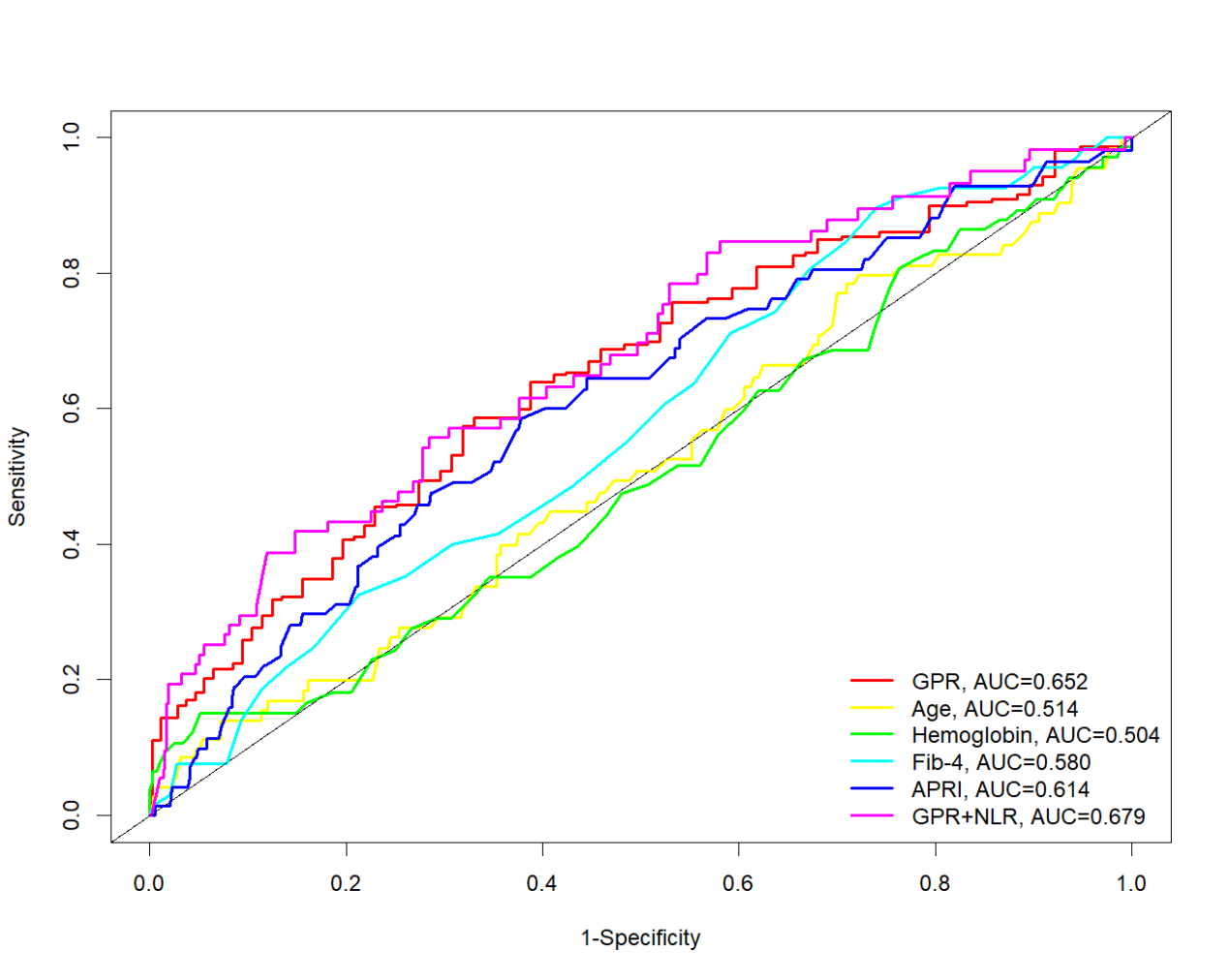


**Figure S2** The ROC curve of GPR, age, hemoglobin, Fib-4, APRI, and the combination of GPR and NLR.

Abbreviation: GPR, gamma-glutamyl transpeptidase-to-platelet ratio; Fib-4, fibrosis 4 score; APRI, aspartate transaminase to platelet ratio index; NLR, neutrophil to lymphocyte ratio index.


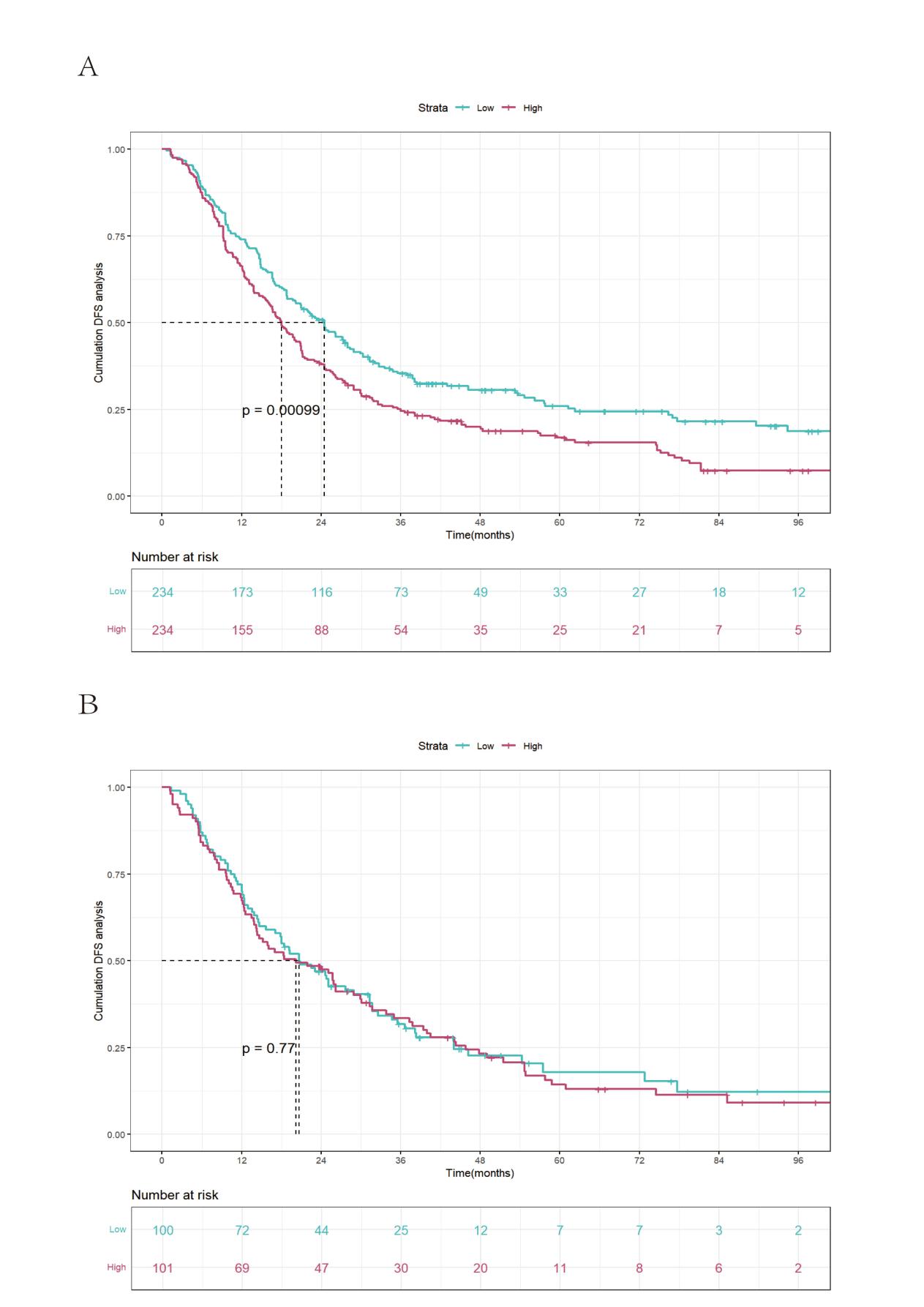


**Figure S3** Kaplan-Meier curves of RFS for two risk groups classified by the nomogram in training and validation cohort. (A) training cohort; (B) validation cohort.

Abbreviation: RFS, recurrence-free survival.
